# Trends in adoption of extravascular cardiac implantable electronic devices: the Dutch cohort

**DOI:** 10.1007/s12471-024-01892-6

**Published:** 2024-08-19

**Authors:** Karel T. N. Breeman, Reinoud E. Knops, Michelle D. van der Stoel, Lucas V. A. Boersma, Sing-Chien Yap, Lieselot van Erven, Vincent F. van Dijk, Alexander H. Maass, Arthur A. M. Wilde, Fleur V. Y. Tjong, Device Registration Committee of the Netherlands Heart Registration

**Affiliations:** 1 Department of Cardiology, Amsterdam UMC location AMC, Amsterdam, The Netherlands; 2 Netherlands Heart Registration, Utrecht, The Netherlands; 3 Department of Cardiology, St Antonius Hospital, Nieuwegein, The Netherlands; 4 Department of Cardiology, Erasmus MC, Cardiovascular Institute, Thorax Center, Rotterdam, The Netherlands; 5 Department of Cardiology, Leiden University Medical Center, Leiden, The Netherlands; 6 Department of Cardiology, University Medical Center Groningen, Groningen, The Netherlands; 7 Heart Failure & Arrhythmias, Amsterdam Cardiovascular Sciences, Amsterdam, The Netherlands

**Keywords:** Extravascular, Leadless pacemaker, Subcutaneous ICD, Adoption, Registration

## Abstract

**Introduction:**

Conventional implantable cardioverter-defibrillators (ICDs) and pacemakers carry a risk of pocket- and lead-related complications in particular. To avoid these complications, extravascular devices (EVDs) have been developed, such as the subcutaneous ICD (S-ICD) and leadless pacemaker (LP). However, data on patient or centre characteristics related to the actual adoption of EVDs are lacking.

**Objective:**

To assess real-world nationwide trends in EVD adoption in the Netherlands.

**Methods:**

Using the Netherlands Heart Registration, all consecutive patients with a de novo S‑ICD or conventional single-chamber ICD implantation between 2012–2020, or de novo LP or conventional single-chamber pacemaker implantation between 2014–2020 were included. Trends in adoption are described for various patient and centre characteristics.

**Result:**

From 2012–2020, 2190 S‑ICDs and 10,683 conventional ICDs were implanted; from 2014–2020, 712 LPs and 11,103 conventional pacemakers were implanted. The general use has increased (S-ICDs 8 to 21%; LPs 1 to 8%), but this increase seems to have reached a plateau. S‑ICD recipients were younger than conventional ICD recipients (*p* < 0.001) and more often female (*p* < 0.001); LP recipients were younger than conventional pacemaker recipients (*p* < 0.001) and more often male (*p* = 0.03). Both S‑ICDs and LPs were mainly implanted in high-volume centres with cardiothoracic surgery on-site, although over time S‑ICDs were increasingly implanted in centres without cardiothoracic surgery (*p* < 0.001).

**Conclusion:**

This nationwide study demonstrated a relatively quick adoption of innovative EVDs with a plateau after approximately 4 years. S‑ICD use is especially high in younger patients. EVDs are mainly implanted in high-volume centres with cardiothoracic surgery back-up, but S‑ICD use is expanding beyond those centres.

**Supplementary Information:**

The online version of this article (10.1007/s12471-024-01892-6) contains supplementary material, which is available to authorized users.

## What’s new?

- EVDs are being increasingly used in the Netherlands and especially S‑ICDs currently constitute a substantial part of device therapy.
- The relative adoption of S‑ICDs is especially high in younger patients, with the highest use in patients aged between 10–40 years.
- EVDs are mainly implanted in high-volume centres with cardiothoracic surgery back-up, but S‑ICD use is spreading beyond centres with cardiothoracic surgery.

## Background

For patients with cardiac arrhythmias, cardiac implantable electronic devices (CIEDs) are an important therapy aimed to improve symptoms and mortality. Conventional CIEDs consist of a subcutaneously implanted generator with an electrode through the veins, which is connected with the myocardium. However, conventional CIEDs are associated with a risk of complications, which are mainly caused by the lead and/or pocket [1, 2]. To overcome those complications, CIEDs have been designed without a lead through the vasculature and, when feasible, also without a subcutaneous pocket. The first commercially available extravascular devices (EVDs) were the subcutaneous implantable cardioverter defibrillator (S-ICD), consisting of a subcutaneous defibrillator with a fully subcutaneous lead, and the leadless pacemaker (LP), a small pacemaker fully contained in the right ventricle. Both have proven to be safe and effective therapies [3, 4]. However, data on patient and centre characteristics related to the adoption of EVDs are lacking. In the Netherlands, EVD therapy was adopted early on, enabling us to study its use from the beginning [5, 6] Therefore, the goals of this study were to describe in a Dutch cohort: (1) the general adoption of EVDs over time; (2) the adoption of EVDs related to patient characteristics; and (3) the adoption of EVDs related to centre characteristics.

## Methods

### Design and data source

This study used prospectively collected cohort data registered at the Netherlands Heart Registration (NHR). The NHR is a quality and registration organisation that receives patient, procedural and follow-up data of all cardiac interventions and surgeries in 72 Dutch hospitals. The data are provided by the data managers of the hospitals. The goal of the NHR is to maintain and improve cardiac care in the Netherlands by collecting, analysing and providing evaluations of data regarding the treatment of patients with heart disease [7]. The use of NHR data for this study was approved by the Medical Research Ethics Committees United (Nieuwegein, the Netherlands).

### Patients

All consecutive patients with a de novo S‑ICD or conventional single-chamber ICD implantation from 1 January 2012 to 31 December 2020, or a de novo LP or conventional single-chamber pacemaker (PM) implantation from 1 January 2014 to 31 December 2020 were included. We used these timeframes to ensure that all EVD registrations were included, as the first S‑ICD was registered at the NHR in 2012 and the first LP in 2014.

The EVDs included in this study were S‑ICDs (Boston Scientific Corporation, St. Paul, MN, USA) and LPs (Nanostim, St. Jude, St. Paul, MN, USA; now Abbott Medical Inc., Abbott Park, IL, USA; Micra VR and Micra AV, Medtronic, Minneapolis, MN, USA). The Nanostim and Micra VR are single-chamber ventricular pacing and sensing (VVI[R]) pacemakers, whereas the Micra AV is a ventricular single-chamber pacing and dual-chamber sensing (VDD[R]) pacemaker.

### Characteristics of patients and implanting centres

The NHR provided data on patient age at implantation and gender. Implanting centres were categorised by mean annual CIED implantation volume during the study period (tertiles; low-volume, range 55–114 implantations per year; middle-volume, range 114–335 implantations per year; high-volume, range 338–1017 implantations per year), type of implanting centre (with or without cardiothoracic surgical back-up) and region (North, Middle or South of the Netherlands). Hence, the adoption of EVDs is described for these patient and implanting centre characteristics. Implausible values were considered missing for this analysis.

### Statistical analysis

Continuous data are described as mean and standard deviation or median and interquartile range, categorical data as number and percentage. Differences between patients with EVDs and non-EVDs and differences between implanting centres of EVDs and non-EVDs were tested for by Student’s T‑test, Chi-square test for trend and Fisher’s exact test. Trends in adoption over time were calculated with linear and logistic regression analysis.

## Results

### General adoption

**Fig. 1:**
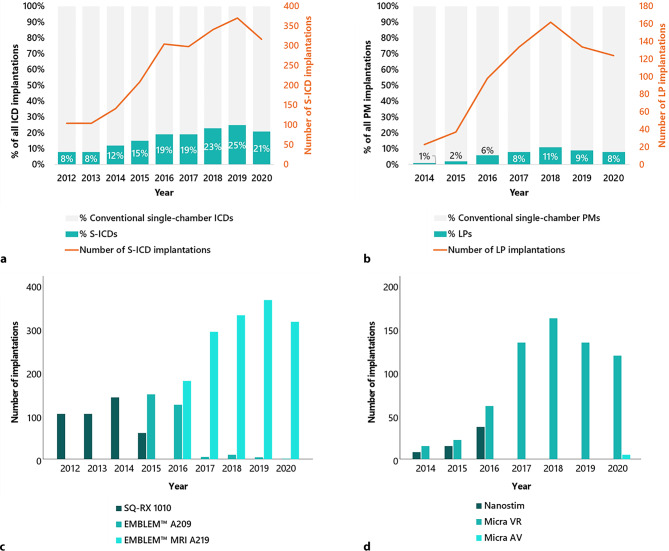
Trends in adoption of S‑ICDs (**a**) and LPs (**b**) among target populations over time, and use of different S‑ICD (**c**) and LP (**d**) models over time

From 2012–2020, a total of 2190 de novo S‑ICDs and 10,683 de novo conventional single-chamber ICDs were implanted. The relative use of S‑ICDs within all included ICDs increased from 8% in 2012 to 21% in 2020 (*p* < 0.001; Fig. 1a). From 2014–2020, a total of 712 de novo LPs and 11,103 de novo conventional single-chamber PMs were implanted. The relative use of LPs within all included PMs increased from 1% in 2014 to 8% in 2020 (*p* < 0.001; Fig. 1b).

Of the S‑ICDs, 410 (19%) were model SQ-RX 1010, 294 (13%) EMBLEM™ A209 and 1486 (68%) EMBLEM™ A219. Of the LPs, 60 (8%) were Nanostim LPs, 647 (91%) Micra VR LPs and 5 (1%) Micra AV LPs. Thus, 707 (99%) VVI(R) LPs were implanted and 5 (1%) VDD(R) LPs. The use of different S‑ICD and LP models over time is shown in Fig. 1c and d.

### Adoption related to patient characteristics

**Fig. 2:**
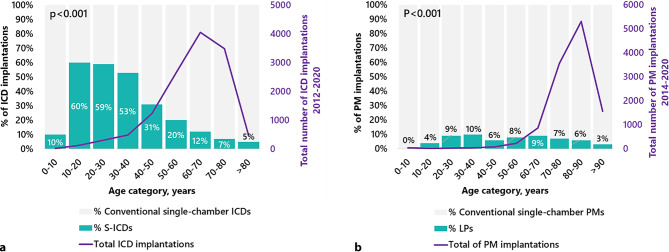
Adoption of S‑ICDs (**a**) and LPs (**b**) among different age categories

S‑ICD recipients were younger at implantation than conventional ICD recipients (52 ± 16 vs. 64 ± 12 years, *p* < 0.001). LP recipients were younger at implantation than conventional pacemaker recipients (78 ± 10 vs. 80 ± 11 years, *p* < 0.001). Mean age at S‑ICD implantation decreased slightly over time (*p* = 0.02) while the mean age at LP implantation was constant (*p* = 0.92). The relative adoption of S‑ICDs was more than half of all included ICDs in the age groups of 10–20, 20–30 and 30–40 years, while for LPs there was no specific subgroup with a higher adoption (Fig. 2).

S‑ICD recipients were, in comparison to conventional ICD recipients, more likely to be female (25% vs. 22%; *p* < 0.001). LP recipients were more likely to be male than conventional single-chamber PM recipients (37% female vs. 41% female, *p* = 0.03). The gender distribution did not change over time for S‑ICD recipients (*p* = 0.60) and LP recipients (*p* = 0.22).

### Adoption related to centre characteristics

**Fig. 3:**
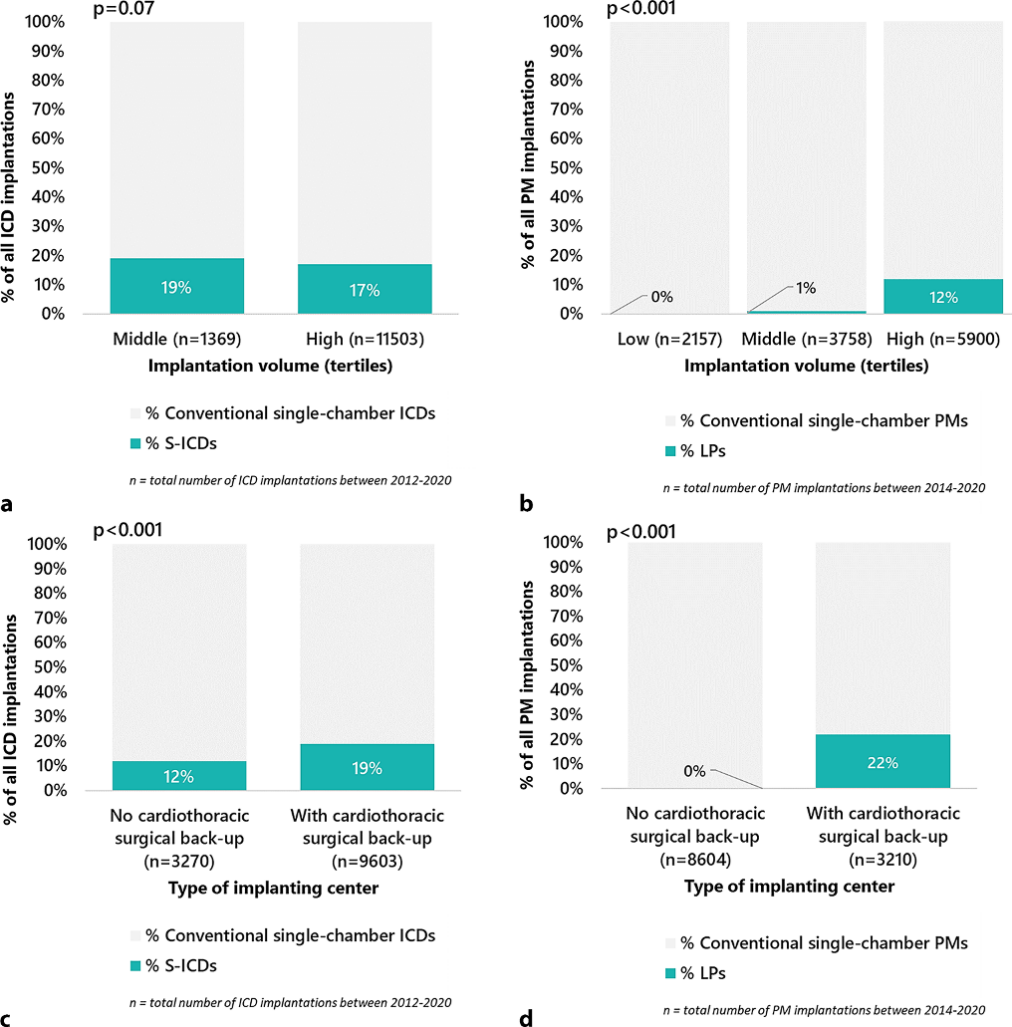
Adoption of EVDs among centres with different device implantation volumes (S-ICDs (**a**), LPs (**b**)) and different types of implanting centre (S-ICDs (**c**), LPs (**d**))

S‑ICDs were only implanted in centres with middle and high device implantation volumes, similarly to conventional single-chamber ICDs. Of all S‑ICDs, 12% were implanted in middle-volume centres and 88% in high-volume centres, with an increasing proportion of implantations in high-volume centres (*p* = 0.002) over time. The relative adoption of S‑ICDs was similar in middle- and high-volume centres (*p* = 0.07; Fig. 3a). LPs were also only implanted in centres with middle and high device implantation volumes (3.5 and 96.5% of LP implantations, respectively, a distribution that was stable over time [*p* = 0.40]), whereas conventional single-chamber PMs were implanted in low-, middle- and high-volume centres. The relative adoption of LPs was lower in middle than in high-volume centres (*p* < 0.001; Fig. 3b).

S‑ICDs were implanted both in centres with and without cardiothoracic surgical back-up (83 and 17% of S‑ICD implantations, respectively). Over time, the proportion of S‑ICDs implanted in centres without cardiothoracic surgical back-up among all implanted S‑ICDs increased from 9% in 2012 to 17% in 2020 (*p* < 0.001) (Fig. S1, in the Electronic Supplementary Material). The relative adoption of S‑ICDs was highest in centres with cardiothoracic surgical back-up (*p* < 0.001; Fig. 3c). LPs were solely implanted in centres with cardiothoracic back-up, where a relative adoption was seen of 22% (Fig. 3d).

The adoption of EVDs differed significantly between regions in the Netherlands. The adoption of S‑ICD out of all included ICDs was 19% in the Northern region, 17% in the Central region, and 10% in the Southern region of the Netherlands (*p* < 0.001). The adoption of LPs out of all included PMs was 6% in the Northern region, 4% in the Central region, and 2% in the Southern region (*p* < 0.001).

## Discussion

This study has three main findings: 1) innovative EVDs were adopted relatively quickly in the Netherlands, and especially the S‑ICD has become a substantial part of device therapy in the Netherlands; 2) utilisation of S‑ICDs was highest among younger patients, while use of LPs was not higher in specific age groups; 3) EVDs were mainly implanted in high-volume centres with cardiothoracic surgery available, although S‑ICDs are increasingly being implanted in centres without cardiothoracic surgery on-site, potentially leading to a broader adoption than LPs in the Netherlands.

This study with nationwide registration data demonstrated that the use of EVDs has been increasing in the Netherlands since their introduction and that S‑ICDs in particular are currently used in a substantial proportion of patients. This study highlights that patients aged 10–40 years with a single-chamber ICD indication form a subgroup with a very high S‑ICD adoption. More than half of those patients received an S‑ICD. Studies from other countries show a similar adoption, including an almost identical distribution of S‑ICD use over age groups in a Japanese cohort [8, 9]. Implanters often choose S‑ICDs in younger patients due to their increased risk of lead-related complications because of their more active lifestyle and longer need for ICD therapy [10]. Also, younger patients rarely have a concomitant indication for antibradycardia, antitachycardia or cardiac resynchronisation therapy pacing. The adoption of LPs was not higher in patients of specific age groups. The finding that LP recipients were slightly younger than conventional single-chamber PM recipients should be put in perspective, as the studied PM population is relatively old compared with the vastly larger group of dual-chamber PM recipients [11]. We believe that among all PM recipients, older patients are generally favoured for LP therapy, due to the current absence of dual-chamber LPs and probably also due to the uncertain end-of-life strategy of LPs, either replacement or abandonment. The relatively higher adoption of S‑ICDs among female patients was also seen in a US database [8].

S‑ICDs were numerically mostly implanted in high-volume centres, but middle- and high-volume centres showed an equal adoption with S‑ICD implantations in approximately one in six patients with a single-chamber ICD indication. This difference in use related to implantation volume might be explained by a higher percentage of younger patients who more often have genetic or congenital heart disease for which patients are typically referred to specialised centres. However, an increasing adoption over time beyond centres with cardiothoracic surgical back-up was demonstrated. Most LP implantations were in high-volume centres, where the use of LPs was highest. The presumable explanation is that the high-volume centres include more centres with cardiothoracic surgery on-site, which is mandatory for LP implantation in the Netherlands. There were significant differences in the adoption of EVDs between regions in the Netherlands, with a two- or three-times higher use of EVDs in the Northern region compared with the Southern region. This may potentially be caused by the centre types in each region and whether centres engaged early on with this technology in clinical trials.

In the most recent years, it can be noted that the growth of EVD use has stopped in the Netherlands. This may be due to temporal causes with the covid-19 pandemic in 2020 and its logistic difficulties. However, the stagnation may potentially also be due to structural causes, such as higher costs of EVDs (approximately factor 3–4 higher). Further, LP implantation is restricted to centres with cardiothoracic surgery available, as perforations occur in ~1% of implantations and require emergency surgery in an estimated quarter of those [12–15]. On the other hand, a further increase in the use of EVDs is expected due to additional experience and broadening of the indication areas using novel EVDs. The main current limitation of S‑ICDs is their lack of pacing capabilities. However, in February 2023, the extravascular ICD (EV-ICD; Medtronic, Minneapolis, MN, USA), which is capable of antitachycardia pacing and back-up pacing through a substernal lead, received Conformité Européenne (CE) approval [16]. Further, a modular system consisting of a communicating S‑ICD and antitachycardia pacing-enabled LP is currently being studied in the first clinical trial [17, 18]. Cardiac resynchronisation therapy using a subcutaneous pulse generator and left ventricular LP is also being studied clinically [19]. The guideline-directed indication area for LPs is expected to expand too. The Micra AV, capable of atrioventricular synchronous pacing, was CE-approved in June 2020 and only the first cases in the Netherlands are described in this study. A clinical trial with a dual-chamber LP, the Aveir DR (Abbott, Medical Inc., Abbott Park, IL, USA), demonstrated adequate results and has already received approval from the Food and Drug Administration [20]. Lastly, the use of LPs may also be influenced by the emergence of conduction system pacing, which has different benefits than LPs. At the moment, patients may simultaneously be good candidates for leadless and conduction system pacing, and a choice should be made based on individual characteristics. Yet, novel LP designs that can pace the conduction system are being studied. To improve the data collection regarding all EVDs including novel devices, a Dutch nationwide registry with long-term follow-up of EVDs will be incorporated in NHR’s device registry [21].

This study was limited by the lack of data after 2020. Therefore, not all currently available EVD models are described. Also, within the conventional single-chamber pacemakers, we could not distinguish between AAI(R) and VVI(R) pacemakers. However, we do think that this group mainly consists of VVI(R) pacemakers as AAI(R) pacemakers are rarely implanted in the Netherlands [1]. Also, no distinction could be made between transvenous and abdominal CIEDs, although the large majority of conventional CIEDs described in this study are expected to be transvenous CIEDs. Lastly, the data presented are purely descriptive and therefore, do not infer causality.

## Conclusion

In this nationwide study we observed a relatively quick adoption of innovative EVDs with a plateau after approximately 4 years. The use of EVDs was higher in younger patients, which was especially true for S‑ICDs. This is likely related to the benefit of avoiding the higher life-time risk of lead-related complications in young people with an S‑ICD. The adoption of LPs in the Netherlands was less in comparison to S‑ICDs, and this may be because LPs can only be implanted in centres with cardiothoracic surgical back-up. Monitoring of the adoption of EVDs remains necessary as the indication area for EVDs is expected to grow.

## Supplementary Information

Device registration committee of the Netherlands Heart Registration

Supplementary Figure 1. Type of implanting center (with or without cardiothoracic surgery back-up) of S‑ICDs, trend over time
