# Supplementary material for: Trends in adoption of extravascular cardiac implantable electronic devices: the Dutch cohort
Source: Neth Heart J. 2024 Aug 19;32(10):356–62. doi: 10.1007/s12471-024-01892-6 (PMC11413308; doi:10.1007/s12471-024-01892-6)
Supplement: Supplementary file 1 — Device registration committee of the Netherlands Heart Registration [file 12471_2024_1892_MOESM1_ESM.docx]

**Device registration committee** **of the Netherlands Heart Registration**

Dr. L.J.P.M. van Woerkens, Albert Schweitzer Hospital

Dr. C.J.H.J. Kirchhof, Alrijne Hospital

Drs. W. Kuijt, Amphia Hospital

Prof. J.R. de Groot, Amsterdam UMC, location AMC

Dr. V.P. van Halm, Amsterdam UMC, location VUMC

Dr. R.M.A. van de Wal, Bernhoven

Dr. I.R. Henkens, Bravis Hospital

Dr. F. Bracke, Catharina Hospital

Dr. J.J. Wiersma, Dijklander Hospital

Dr. H.J. Muntinga, Elisabeth-TweeSteden Hospital

Drs. R.E. Bhagwandien, Erasmus Medisch Centrum

Drs. S.D.A. Valk, Franciscus Gasthuis & Vlietland

Dr. A.M. Otten, Gelre Hospitals

Dr. R.W. Grauss, Haaglanden Medical Center

Dr. H. Ramanna, Haga Hospital

Drs. J.J.H. Bennik, IJsselland ziekenhuis

Dr. A. Adiyaman, Isala Hospital

Drs. R. Joustra, Jeroen Bosch Hospital

Dr. F.M. Hörters, Maasstad Hospital

Dr. J.G.L.M. Luermans, Maastricht UMC+

Drs. M. Smit, Martini Hospital

Dr. T. Vromen, Máxima MC

Drs. E.A. de Vrey, Meander MC

Dr. B.A. Schoonderwoerd, Medical Center Leeuwarden

Dr. J.M. van Opstal, Medisch Spectrum Twente

Dr. G.P. Kimman, Noordwest Ziekenhuisgroep

Dr. I.E. Hof, OLVG

Drs. E.P. Meindersma, Radboudu

Dr. F.P.J. Brouwers, Rijnstate

Dr. A.B. van Zoelen, Beatrix Hospital

Dr. I.C.D. Westendorp, Rode Kruis Hospital

Drs. B.M. van Bemmel, Saxenburgh Medical Center

Dr. V.F. van Dijk, St. Antonius Hospital

Dhr. W.P.J. Jansen, Tergooi MC

Dr. A.E. Tuinenburg, University Medical Center Utrecht

Dr. A.H. Maass, Universitair Medisch Centrum Groningen

Drs. R. Hazeleger, VieCuri Medical Center

Dr. M.W.Z. Basalus, Ziekenhuisgroep Twente

Drs. B. Broers, Zuyderland Medical Center
