# Supplementary material for: Trends in adoption of extravascular cardiac implantable electronic devices: the Dutch cohort
Source: Neth Heart J. 2024 Aug 19;32(10):356–62. doi: 10.1007/s12471-024-01892-6 (PMC11413308; doi:10.1007/s12471-024-01892-6)
Supplement: Supplementary file 2 — Supplementary Figure 1. Type of implanting center (with or without cardiothoracic surgery back-up) of S‑ICDs, trend over time [file 12471_2024_1892_MOESM2_ESM.docx]

**Supplementary Figure 1.** Type of implanting center (with or without cardiothoracic surgery back-up) of S-ICDs, trend over time.

**
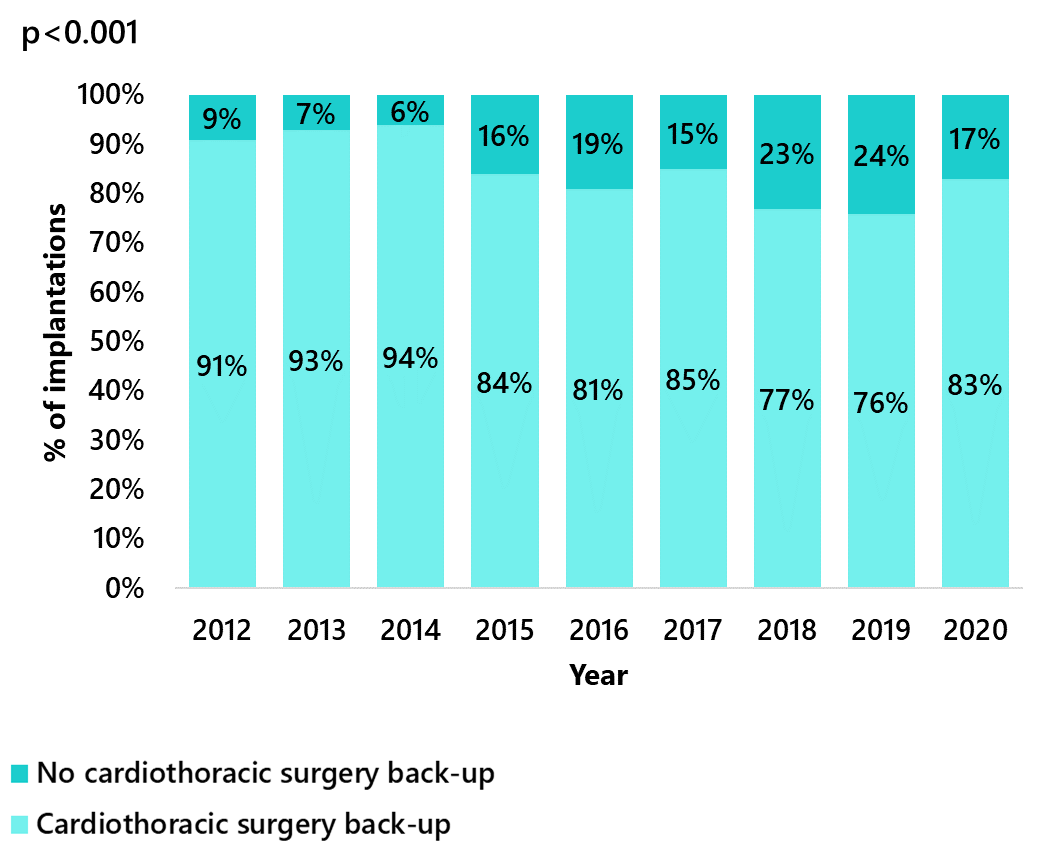
**
